# Age-associated alterations in γδ T-cells are present predominantly in individuals infected with Cytomegalovirus

**DOI:** 10.1186/1742-4933-10-26

**Published:** 2013-07-03

**Authors:** Kilian Wistuba-Hamprecht, Daniela Frasca, Bonnie Blomberg, Graham Pawelec, Evelyna Derhovanessian

**Affiliations:** 1 Department of Internal Medicine II, Centre for Medical Research, University of Tübingen, Tübingen, Germany; 2 Department of Microbiology and Immunology, University of Miami Miller School of Medicine, Miami, FL, USA

**Keywords:** γδ T-cells, Differentiation phenotype, CMV, Human ageing

## Abstract

**Background:**

Despite the common perception that latent Cytomegalovirus (CMV) infection is usually symptom-free, emerging epidemiological evidence suggests that it may in fact be associated with higher mortality over extended follow-up. Mechanisms responsible for this potentially important effect are unclear. CMV infection is known to have a large impact on the distribution of T cell phenotypes, especially the accumulation of late-stage differentiated CD8^+^, as well as Vδ2^-^ γδ T-cells, which are the main subset of γδ T-cells involved in anti-CMV immunity. Its impact on γδ T-cells in the aging context is less well-defined.

**Results:**

Here, we investigated a group of healthy individuals aged between 21 and 89 years, in order to correlate the frequency and differentiation status of γδ T-cells with age. We found that these parameters were only marginally influenced by age, but were marked in people with a latent CMV infection. Thus, we observed a significant age-associated accumulation of late-differentiated T-cells within the Vδ2^-^ population, but only in CMV-seropositive donors. There was also a strong trend towards reduced frequency of early-differentiated cells within the Vδ2^-^ phenotype. Older people had significantly higher anti-CMV IgG titers, which in turn correlated significantly with a lower Vδ2^+^/Vδ2^-^ ratio and a shift from early- to a late-differentiated Vδ2^-^ T-cell phenotype.

**Conclusions:**

Our findings demonstrate a strong influence of CMV on γδ T-cells during human ageing, similar to that observed for αβ T-cells. Differences between donors of different ages are more marked in CMV-infected individuals. The biological implications of this potent age-associated CMV-mediated immune-modulation require clarification.

## Background

γδ T-cells represent a minor population of approx. 5% of the whole T-cell population in healthy adult individuals. The ligands recognized by the γδ receptor are yet not fully identified, but it is known that different isotypes of γδ T-cell receptor (TCR) recognize common stress-induced molecules on self cells as well as structures on the surface of pathogens. The majority of γδ T-cells is CD4^-^CD8^-^, but a small population expresses CD8 [1]. γδ T-cells are further grouped via their different δ-chains. The majority (approximately 70%) of γδ T-cells in human blood is represented by the Vδ2 receptor. The remaining γδ T-cells in the blood mainly express the Vδ1 receptor but other more rare subpopulations such as Vδ3, Vδ4 or Vδ5 have also been described. Vδ2^+^ cells recognize nonpeptidic phosphorylated metabolites of isoprenoid biosynthetic pathways produced by a variety of bacteria, parasites and some tumor cells, whereas Vδ1 T cells are activated by stress-induced molecules such as MIC-A and MIC-B [2,3].

Several investigations into the phenotypic and functional alterations of γδ T-cells in the human aging process have been initiated in the last few years, some demonstrating a decrease in γδ T-cell numbers with age, mainly due to declining numbers of the Vδ2^+^ subset, with the Vδ1 population remaining stable [4-8]. There is also one publication demonstrating a decrease in naïve and early differentiated cells and an increase in late differentiated γδ T-cells in men but not in women [7]]. Many similar age-associated changes described in αβ T-cells, particularly the accumulation of late-differentiated CD8 T-cells, have been attributed to a latent infection with human Cytomegalovirus (CMV) and not chronological age [9-11]. CMV is a ubiquitous beta-herpes virus causing mostly asymptomatic infection in healthy individuals. After the first infection, the virus enters cellular latency, but can reactivate repeatedly under different conditions [12]. Both CD4 and CD8 αβ T-cells are involved in anti-CMV immune surveillance. In fact a large fraction of these cells can be specific for this single virus [13], which can lead to an accumulation of late-differentiated CD8 T-cells, reciprocated by a decrease in naïve T-cells [14]. γδ T-cells have also been implicated in anti-CMV immunity and control of infection [15-18] which is mediated through Vδ2^-^ cells (mainly Vδ1 and Vδ3 but also Vδ5 [15,18]). Accordingly, CMV-seropositivity has been associated with higher frequencies and absolute numbers of Vδ2^-^ cells in the periphery [4,17,19-21] and with a more restricted repertoire compared to Vδ2^+^ cells [19].

The proportion of the population infected with CMV increases steadily with age in Western countries with a seroconversion rate of 1-3% per year [22] and has been associated with higher morbidity and mortality in some studies [23,24]. Whether a latent infection with this virus has any impact on possible age-associated putative changes to the phenotype of γδ T-cells, as is the case with αβ T-cells, has not been explored before. Here, we present data demonstrating that similar to αβ T-cells, age-associated differences in γδ T-cell frequency and differentiation phenotype are predominantly present in individuals with a latent infection with CMV, but in uninfected people. Additionally, we describe a negative correlation between a more late-differentiated phenotype of Vδ2^-^ cells and anti-CMV IgG levels in serum, which increased significantly with age in our study cohort.

## Results

### Minor impact of age on the frequency and phenotype of γδ T-cells in the absence of CMV infection

**Figure 1:**
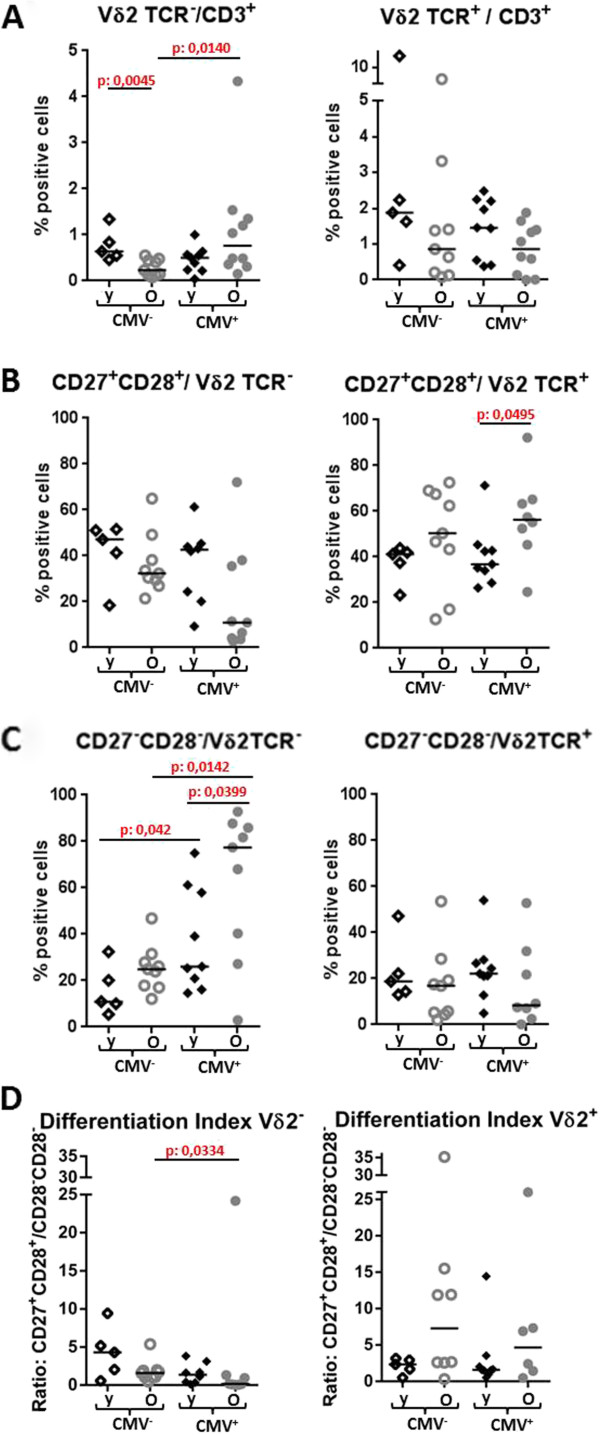
**Impact of CMV-serostatus on frequency and differentiation status of γδ T-cells.** The frequency of Vδ2^-^ and Vδ2^+^ γδ T-cells within total T-cells **(A)**, CD27^+^CD28^+^ early-differentiated **(B)** and CD27^-^CD28^-^ late-differentiated cells **(C)** within Vδ2^+^ and Vδ2^-^ cells as well as the differentiation index (CD27^+^CD28^+^/CD27^-^CD28^-^) for each subset **(D)** were compared between CMV-seronegative (CMV^-^) and CMV-seropositive (CMV^+^) individuals between 21 and 89 years of age. Each symbol represents a single donor. Horizontal bars represent the median of the group. P values were calculated using the Mann-Whitney U-test. Y: young 21 to 38 years, 10 female, 4 male; O: old, 55 to 89 years, 11 female, 8 male).

Given the strong impact of CMV-seropositivity on age-associated alterations observed in αβ T-cells, we sought to determine if the same was true for γδ T-cells. For this, individuals were grouped according to CMV-serostatus and age, and the frequency and phenotype of γδ T-cells was compared. In CMV-seropositive individuals we observed a significantly lower ratio of Vδ2^+^/Vδ2^-^ cells in older individuals (Figure 1A, left-hand panel), as well as a strong age-associated trend towards a reduction in early-differentiated CD27^+^CD28^+^ Vδ2^-^ T-cells (p=0.059) reciprocated by a significant accumulation of CD27^-^CD28^-^Vδ2^-^ T-cells (p=0.0399) (Figure 1B and C, left-hand graphs). Contrary to the Vδ2^-^ cells, in the Vδ2^+^ subset, ageing was associated with higher frequency of early-differentiated cells (Figure 1B, right-hand panel, p=0.049). These differences were absent in CMV-seronegative donors (Figure 1). Here, we only observed a significant reduction of Vδ2^-^ T-cells (Figure 1A left-hand graph, p=0.0045).

### CMV-seropositivity is associated with phenotypic differences in γδ T-cells

Having established a strong impact of CMV on the manifestation of age-associated changes in the phenotype and frequency of γδ T-cells, we investigated the impact of a latent infection with CMV on these parameters in young and old CMV-seronegative and CMV-seropositive individuals. We observed a significantly higher proportion of Vδ2^-^ cells in old CMV-seropositive donors (p=0.014, Figure 1A, left-hand graph). Although the differentiation phenotype of the Vδ2^+^ population was not associated with CMV-seropositivity, strong correlations were observed in the Vδ2^-^ group. Thus, in old CMV-seropositive donors, there was a trend toward lower proportions of early-differentiated cells (Figure 1B, left-hand panel, p=0.0.0934). Significantly higher proportions of late differentiated cells were observed in both young (p=0.042) and old (p=0.0142) CMV-seropositive donors compared to age-matched CMV-seronegative individuals (Figure 1C, left-hand graphs). Accordingly, the differentiation index of Vδ2^-^ cells (the ratio between the two early and late differentiated phenotypes) was lower in CMV-seropositive donors (Figure 1D, left-hand panel, p=0.0334 for the old and p=0.0653 for the young).

**Figure 2:**
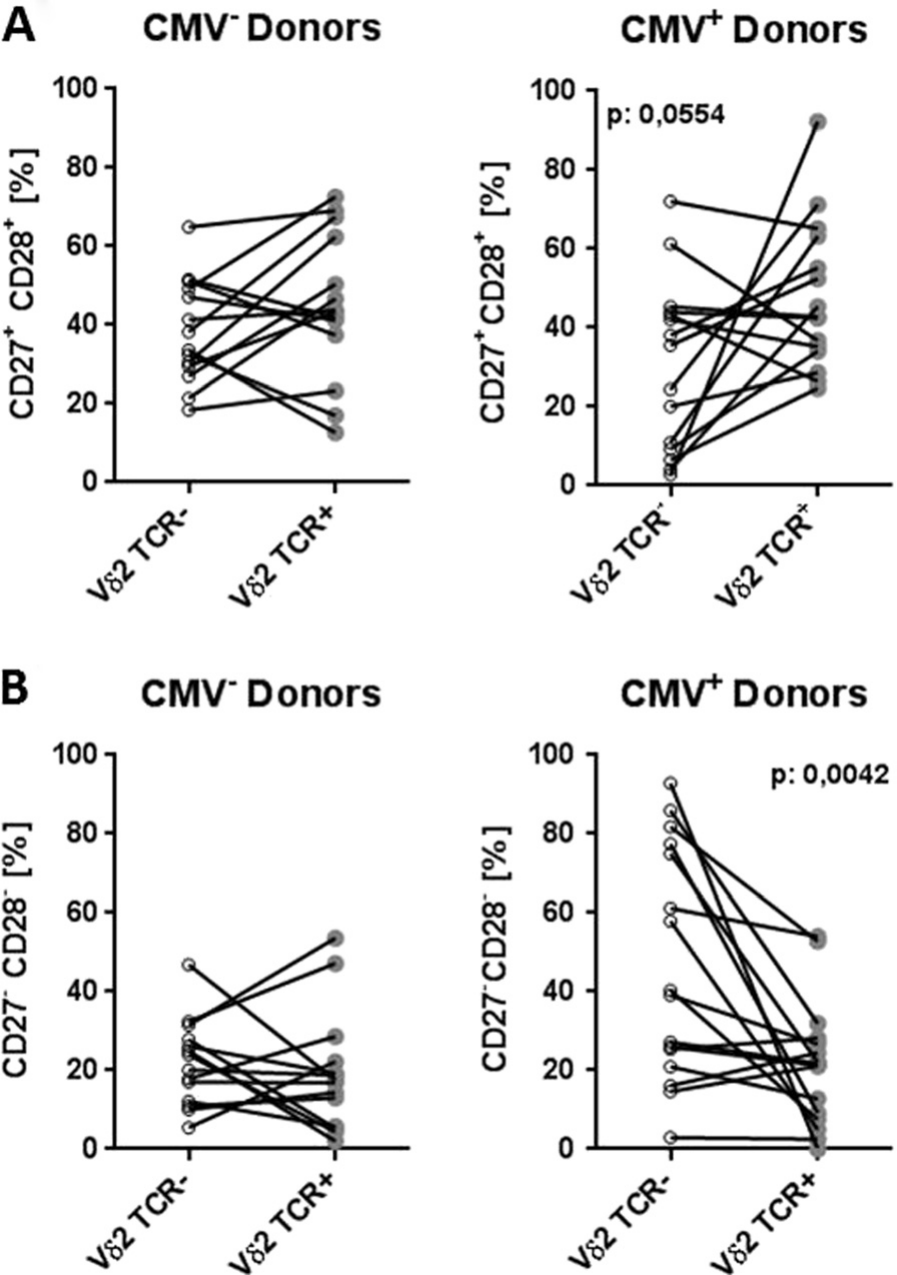
**Inter-individual comparison of the differentiation status of Vδ2**^**- **^**and Vδ2**^**+ **^**cells in individuals stratified according to CMV. A)** frequency of early-differentiated CD27^+^CD28^+^ and **B)** late-differentiated CD27^-^CD28^-^ cells within the Vδ2^-^ and Vδ2^+^ subsets is shown in CMV-seronegative (left-hand panel) and CMV-seropositive (CMV^+^, right-hand) donors between the age of 21 and 89. P values were calculated using the Wilcoxon matched-pairs signed rank test.

Confirming our findings at the population level, we observed a more late-differentiated phenotype of Vδ2^-^ T-cells compared to Vδ2^+^ T-cells within each individual. In the majority of CMV-seropositive donors, the Vδ2^-^ subset contained a lower proportion of the early-differentiated CD27^+^CD28^+^ phenotype (p=0.0554, Figure 2A, right-hand panel) and a significantly higher proportion of the late-differentiated CD27^-^CD28^-^ phenotype (p=0.0042, Figure 2B, right-hand panel) compared to the Vδ2^+^ subsets. This was not observed in CMV-seronegative donors (Figure 2, left-hand panels).

### Anti CMV-IgG titers rise with age and correlate with the differentiation status of γδ T-cells

**Figure 3:**
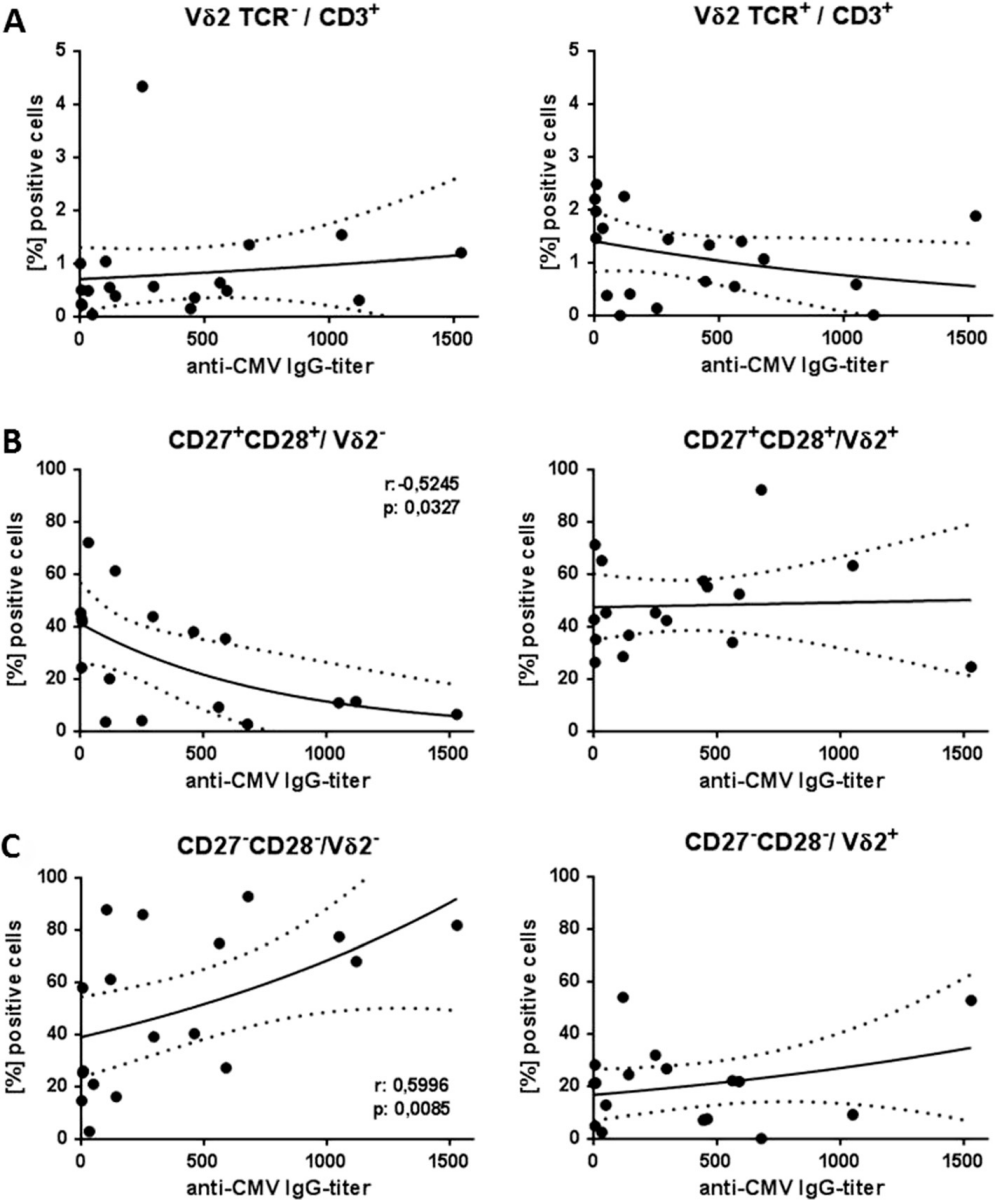
**Correlation between CMV-specific IgG titer and the frequency and differentiation phenotype of Vδ2**^**- **^**and Vδ2**^**+ **^**γδ T-cells.** Frequency of cells lacking or expressing the Vδ2 receptor **(A)** as well as cells with a CD27^+^CD28^+^**B)** or a CD27^-^CD28^-^ phenotype **C)** within Vδ2^-^ (left-hand panel) and Vδ2^+^ (right-hand panel) was correlated with anti-CMV IgG levels in serum of 19 CMV-seropositive individuals between the ages of 21 and 89. P values were calculated using the Spearman correlation test.

In an attempt to elucidate the reason behind a strong impact of CMV on age-associated differences in γδ T-cells, we performed a correlation analysis between age and anti-CMV IgG titers. In our cohort, increasing age was associated with higher levels of anti-CMV IgG in serum (r=0.5320, p=0.019). For this reason and also considering the large heterogeneity of CMV-seropositive donors for some of the variables shown in Figure 1, we asked whether the level of anti-CMV IgG correlated with particular γδ T-cell subsets and phenotypes. We found a significant decrease in the Vδ2^+^/Vδ2^-^ ratio (r=-0.5170, p=0.028) with increasing titer, whereas the latter parameter did not correlate directly with the frequency of cells expressing the Vδ2^+^ receptor or lacking it (Figure 3A). Significant correlations were observed between the differentiation status of Vδ2^-^ T-cells and anti-CMV IgG titer: the latter correlated negatively with the frequency of early differentiated (r= -0.5245, p=0.0327, Figure 3B, left-hand panel) and positively with the frequency of late differentiated (r= 0.5996, p=0.0085, Figure 3C, left-hand panel) Vδ2^-^ T-cells. Accordingly, the differentiation index of Vδ2^-^ cells decreased with rising CMV-specific IgG titers (r=-0.5912, p=0.0288). There was no correlation between the differentiation status of Vδ2^+^ T-cells and CMV-specific IgG titers (Figure 3B and C, right-hand panels).

## Discussion

This study demonstrates for the first time that age-associated differences in γδ T-cell populations are marked only in individuals with a latent CMV infection. Several studies have reported a decrease in the counts and frequencies of γδ T-cells with ageing, which is mainly due to the reduction in the Vδ2^+^ subset [4-8]. We also observed a trend towards age-associated reduction in the frequency of these cells, statistically significant when CMV-seronegative and –seropositive donors were pooled (p=0.01, data not shown). There was also a significant accumulation of cells with a late-differentiated phenotype of the Vδ2^-^ γδ T-cells with increasing age, but only in CMV-seropositive individuals. This is consistent with CMV as a driving force for the accumulation of late-differentiated γδ T-cells, as is the case for CD8^+^ αβ T-cells [9-11]. Those data demonstrating no impact of chronological age on reduction of naïve and accumulation of late-differentiated memory CD8 T-cells in CMV-seronegative individuals, therefore also apply to Vδ2^-^ cells which have been implicated in anti-CMV immunity in several studies [4,15-21]. Thus, the age-associated increase in the differentiation status of these cells in the elderly might be explained by their more frequent encounter with the virus, either due to an earlier time of primary infection and/or more reactivation rounds of the virus, which might take place in the elderly [25]. Since both primary infection and reactivation are usually asymptomatic, it is not possible to determine viral infection and reactivation history with current virological tools. However, we did observe age-associated higher anti-CMV IgG titers in our study, possibly indicating greater activity of the immune system against this virus as an individual ages. To the best of our knowledge, this is the first study to show an age-associated difference in the differentiation status of Vδ2^-^ T-cells, whereas the same trend was not seen in Vδ*2*^+^ cells, which are not involved in anti-CMV immunity. Two studies have reported a decrease in the frequency of naïve-phenotype cells within the Vδ2^+^ subset with increasing age [4,7], although this was reported to be limited to men in one study [7]. The discrepancy between our finding and the published data might be due to the overrepresentation of women in our cohort and the use of different surface markers for phenotyping the differentiation status.

Stratifying the donors according to CMV-serostatus revealed significantly higher frequencies of Vδ2^-^ T-cells in CMV^+^ older individuals, in line with previously published data indicating expansion of Vδ2^-^ cells in healthy carriers of the virus [17,19,20] as well as large expansions of these cells in transplant recipients undergoing primary infection with or reactivation of CMV [15,17,20]. Moreover, Vδ2^-^ cells in CMV-seropositive individuals had a more late-differentiated phenotype compared to CMV-seronegative individuals, in line with previously published data [19,20], whereas the phenotype of Vδ2^+^ cells was not affected by a latent infection with CMV. Confirming these findings at the individual level, Vδ2^-^ cells in CMV-seropositive donors were significantly more late-differentiated compared to Vδ2^+^ cells from the same donor.

Increasing evidence suggests that not only mere seropositivity for the virus, but the way the immune system is affected by the virus and deals with it, may have implications in healthy ageing and mortality [10,26]. Accordingly, not merely the presence of a latent infection with the virus *per se,* but the level of anti-CMV IgG antibodies in serum has been shown to correlate with all-cause mortality in some studies [27,28]. In the present study, we observed a significant correlation between the anti-CMV IgG titer and the differentiation status only of Vδ2^-^ T-cells, whereas again no correlation was observed for the Vδ2^+^ subset. Although similar data are lacking on γδ T-cells, we have previously shown that individuals with higher anti-CMV IgG titers possess significantly higher frequencies and absolute numbers of late-differentiated CD4^+^ T-cells [29]. High levels of anti-CMV IgG antibody might indicate more activation rounds of the virus thus leading to a more late-differentiated phenotype of CMV-reactive Vδ2^-^ γδ T-cells, which may be involved in viral control. Another possible explanation might be a direct stimulation of Vδ2^-^ T-cells through anti-CMV IgG and the virus during reactivation rounds. It was elegantly demonstrated in a recent study that late-differentiated Vδ2^-^ T-cells in CMV-seropositive individuals express high levels of CD16 and can be activated by ligation of this receptor through anti-CMV IgG in the presence of the virus [20]. Vice versa, this correlation might be due to a helper role of γδ T-cells, similar to that of CD4^+^ αβ T-cells in the induction of a humoral immune response - γδ T-cells express essential costimulatory molecules such as CD40L, OX40, CD70 and ICOS, when activated in vitro [30]. Further experiments are needed to elucidate the link between high levels of anti-CMV IgG titers and the more late-differentiated phenotype of γδ T-cells observed in our study.

## Conclusions

The data presented here demonstrate that the strong impact of CMV-infection on T cell phenotypes generally attributed to chronological ageing of the immune system is not observed exclusively in αβ T-cells, but analogously to the greater effect on αβ CD8-vs-CD4 T-cells, is also observed inVδ2^-^ T-cells, in line with the involvement of these cells in anti-CMV immunity.

## Material and methods

### Subjects

Experiments were conducted using blood isolated from healthy volunteers of different ages after appropriate signed informed consent and were approved by the Ethics Commission (IRB protocol #20070481). The individuals participating in this study were screened for diseases known to alter the immune response or for consumption of medications that could alter the immune response. Volunteers were recruited at the University of Miami, Miller School of Medicine. In every experiment reported in the present paper, 33 healthy donors (21 women and 12 men) between the ages of 21 and 89 years (mean age 51.5 years) were evaluated.

CMV antibody screening was performed using cobas 6000® e601 analyzer (Roche, Hitachi) with Elecsys CMV assays (Roche Diagnostics, Mannheim, Germany): CMV IgM (μ-capture assay, cutoff index <0.7; ≥ 1.0, reactive); CMV IgG (cutoff < 0.5 U/ml; reactive ≥ 1..0 U/ml), and CMV IgG avidity (< 45.0 %, low avidity; 45.0-54,9%, grey zone; ≥55,0%, high avidity). In cases of borderline or positive CMV IgM results a recombinant CMV IgM immunoblot (Mikrogen, Neuried, Germany) was performed for confirmation. To ensure that there was no acute CMV-infection at blood donation, here we analysed only donors who were negative for anti-CMV IGM antibody.

### Flow cytometry

After thawing, cryopreserved peripheral blood mononuclear cells (PBMCs) were first treated with human immunoglobulin, GAMUNEX (Bayer, Leverkusen, Germany) and ethidium monoazide bromide (EMA, MoBiTec Gmbh, Göttingen, Germany) in order to block surface Fc receptors and non-viable cells respectively. The cells were then stained indirectly with a monoclonal pan γδ TCR-antibody (clone: 11F2; BD Pharmingen, Heidelberg, Germany) which was detected via Pacific Orange-labelled Fab2’ fragment goat anti-mouse (Live Technologies, Darmstadt, Germany). After blocking with mouse serum (Millipore, Temecula CA, USA), directly conjugated monoclonal antibodies CD3 Alexa Fluor 700 (clone: UCHT-1), CD8 APC-H7 (clone: SK1), CD28 PE (clone: CD28.2) (all from BD Biosciences), CD4 PE-Cy7 (clone: OKT4), CD27 APC (clone: 0323), and anti-Vδ2 TCR PerCP (clone: B6) (all from BioLegend, San Diego, USA) were added for 20 minutes on ice. After washing twice, the cells were measured immediately on an LSR II (BD, Heidelberg). The compensation of the spectral overlap was calculated automatically by BD FACSDiva software, based on the measurement of single colour controls. Data were analysed using FlowJo V 7.6.5 (Tree Star, Portland, USA).

For the analysis, the lymphocyte population was gated in a forward / sideways scatter plot followed by exclusion of duplets in an FSC-height-vs. FSC-width plot. After exclusion of EMA^+^ dead cells, CD4^-^CD8^-^ cells were gated within the CD3^+^ gate and used as starting populations to analyse γδ T-cells, which were characterised as γδ^+^Vδ2^+^ or γδ^+^Vδ2^-^. The latter populations were further studied for CD27 and CD28 expression to determine their differentiation phenotype (see Additional file 1 for the gating strategy). All parental populations <120 counts were excluded from further analysis. Data analysis and flow cytometry staining were performed on blinded samples.

### Statistical analysis

Statistical analysis was performed using GraphPad V6 (GraphPad Software, Inc., La Jolla, USA). For comparisons between two independent groups the Mann-Whitney U Test was used. Correlation between two continuous variables was analyzed using the Spearman-Person Test. Comparisons between two variables within each individual were performed using the Wilcoxon matched-pairs signed rank test. P-values <0.05 were considered significant.

## Abbreviations

CD: Cluster of differentiation; CMV: Cytomegalovirus; EMA: Ethidium monobromide azide; ICOS: Inducible T-cell Costimulator; Ig: Immunoglobulin; TCR: T-cell receptor.

## Competing interests

The authors declare no competing interests.

## Authors’ contributions

KWH was involved in data acquisition, analysis and interpretation as well as drafting the manuscript. GP and ED were involved in designing the experiments, analysis and interpretation of data and drafting the manuscript. DF and BB were involved in recruiting the individuals participating in the study, discussion of the data obtained and writing of the manuscript. All authors have approved the final version.

## Supplementary Material

Additional file 1 **Gating-strategy for γδ T-cell frequency and differentiation phenotype.** Flow cytometry plots representing the gating strategy used for characterisation of γδ T-cells and their differentiation phenotype. Click here for file
