# Supplementary material for: Age-associated alterations in γδ T-cells are present predominantly in individuals infected with Cytomegalovirus
Source: Immun Ageing. 2013 Jul 3;10:26. doi: 10.1186/1742-4933-10-26 (PMC3703274; doi:10.1186/1742-4933-10-26)
Supplement: Additional file 1 — Gating-strategy for γδ T-cell frequency and differentiation phenotype. Flow cytometry plots representing the gating strategy used for characterisation of γδ T-cells and their differentiation phenotype. [file 1742-4933-10-26-S1.pdf]

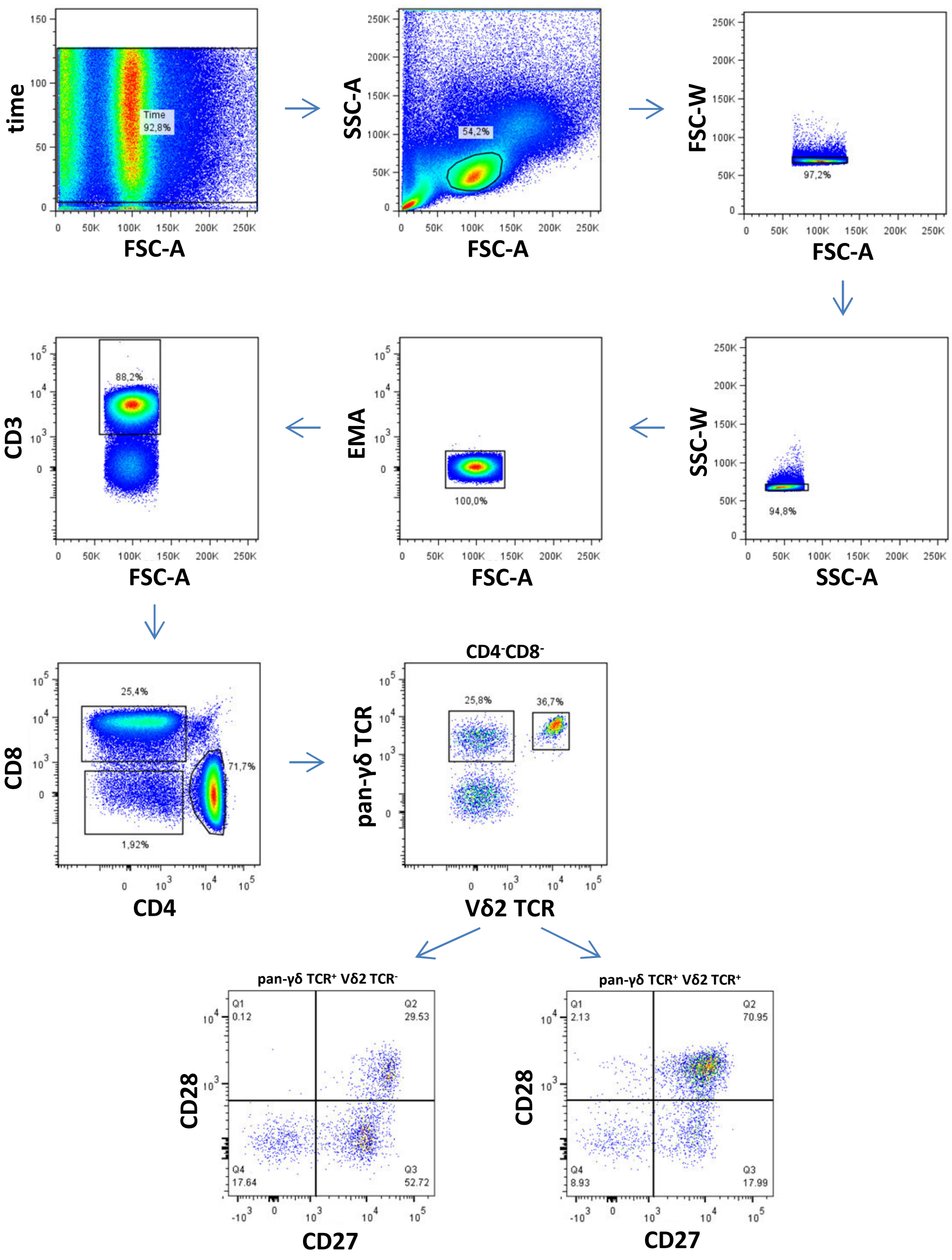

Additional File 1. Gating-strategy for γδ T-cell frequency and differentiation phenotype. After exclusion of events acquired during non-constant flow of the machine in a time vs FSC plot, the lymphocyte population was gated in a FSC vs SSC plot followed by exclusion of duplets in an FSC-height- vs. FSC-width plot. After exclusion of EMA$^+$ dead cells, CD4$^-$ CD8$^-$ cells were gated within the CD3$^+$ gate and used as starting populations to analyse γδ T-cells, which were characterised as γδ$^+$ Vδ2$^+$ or γδ$^+$ Vδ2$^-$. The latter populations were further studied for CD27 and CD28 expression to determine their differentiation phenotype. This strategy was used for analysing all 33 samples.
